# Intrinsic activation of β-catenin signaling by CRISPR/Cas9-mediated exon skipping contributes to immune evasion in hepatocellular carcinoma

**DOI:** 10.1038/s41598-021-96167-0

**Published:** 2021-08-24

**Authors:** Masafumi Akasu, Shu Shimada, Ayano Kabashima, Yoshimitsu Akiyama, Masahiro Shimokawa, Keiichi Akahoshi, Atsushi Kudo, Shoji Yamaoka, Minoru Tanabe, Shinji Tanaka

**Affiliations:** 1 Department of Molecular Oncology, Graduate School of Medicine, Tokyo Medical and Dental University, 1-5-45 Yushima, Bunkyo-ku, Tokyo, 113-8519 Japan; 2 Department of Hepato-Biliary-Pancreatic Surgery, Graduate School of Medicine, Tokyo Medical and Dental University, Tokyo, Japan; 3 Department of Molecular Virology, Graduate School of Medicine, Tokyo Medical and Dental University, Tokyo, Japan

**Keywords:** Hepatocellular carcinoma, Immune evasion

## Abstract

Comprehensive analysis of clinical samples has recently identified molecular and immunological classification of hepatocellular carcinoma (HCC), and the *CTNNB1* (β-catenin)-mutated subtype exhibits distinctive characteristics of immunosuppressive tumor microenvironment. For clarifying the molecular mechanisms, we first established human and mouse HCC cells with exon 3 skipping of β-catenin, which promoted nuclear translocation and activated the Wnt/β-catenin signaling pathway, by using newly developed multiplex CRISPR/Cas9-based genome engineering system. Gene set enrichment analysis indicated downregulation of immune-associated gene sets in the HCC cells with activated β-catenin signaling. Comparative analysis of gene expression profiles between HCC cells harboring wild-type and exon 3 skipping β-catenin elucidated that the expression levels of four cytokines were commonly decreased in human and mouse β-catenin-mutated HCC cells. Public exome and transcriptome data of 373 human HCC samples showed significant downregulation of two candidate cytokine genes, *CCL20* and *CXCL2*, in HCC tumors with β-catenin hotspot mutations. T cell killing assays and immunohistochemical analysis of grafted tumor tissues demonstrated that the mouse *Ctnnb1*^Δex3^ HCC cells evaded immunosurveillance. Taken together, this study discovered that cytokine controlled by β-catenin signaling activation could contribute to immune evasion, and provided novel insights into cancer immunotherapy for the β-catenin-mutated HCC subtype.

## Introduction

Hepatocellular carcinoma (HCC) is a complex disease with various risk factors, that is, chronic infection with hepatitis B virus and hepatitis C virus, alcohol abuse, metabolic disease including obesity and diabetes, and dietary toxins such as aflatoxins and aristolochic acid^1^. Although anti-angiogenic agents and immune checkpoint blockers have currently emerged for HCC treatment^2^, it remains a leading cause of cancer-related death in the world^3^. To improve patient prognosis, categorization of tumor samples into subtypes and customization of cancer therapy for each subtype are essential in HCC, similarly to other types of cancer^4^. Several laboratories have proposed molecular classification of HCC on the basis of gene expression profiles in the past two decades, and a two-group model (proliferation and non-proliferation) is now widely accepted^5,6^. In our latest paper, recent advances in next generation sequencing technology elucidate that the non-proliferation group is further divided into two distinct subtypes, namely *CTNNB1*-mutated and metabolic disease-associated subtypes^7^.

Somatic mutations of *CTNNB1*, encoding β-catenin, are most frequently identified in HCC, and accumulated in exon 3 (amino acid position 5–80) corresponding to the serine/threonine (Ser/Thr) phosphorylation site for GSK3β which normally promotes ubiquitination and degradation of β-catenin. Gain-of-function mutations in exon 3 or exon 3 skipping events contribute to stabilization, translocation from cytoplasm to nucleus, and then activation of the Wnt/β-catenin signaling pathway^8,9^. For examining this biological process, two genetically engineered mouse models have been developed; one is a transgenic mouse model with ectopic expression of mutated and stabilized β-catenin in which Ser33, Ser37, Thr41 and Ser45 are substituted by alanine residues^10^ or N-terminal deletion^11^; the other is a *Cre*/*loxP*-based mouse model harboring a mutant *Ctnnb1* allele with *loxP* sequences in intron 2 and intron 3 for intrinsically skipping of exon 3^12^.

A series of studies has linked tumor-intrinsic Wnt/β-catenin signaling not only to oncogenesis and stemness, but also to cancer immune surveillance. T cell-inflamed phenotype, characterized by CD8+ T cell infiltration, is closely correlated with the efficacy of immune checkpoint blockade, whereas non-T cell-inflamed tumors rarely benefit. Luke et al*.* have recently addressed that the Wnt/β-catenin signaling pathway is activated, particularly by *CTNNB1* mutation, in non-T cell-inflamed tumors across cancer types including HCC^13^. In melanoma, β-catenin signaling upregulates IL-10 secretion, which impairs the capacity of dendritic cells (DCs) to cross-prime CD8+ cytotoxic T cells^14^, or downregulates CCL4 expression, resulting in DC defective recruitment and T cell exclusion^15^. However, although we and others have reported that *CTNNB1*-mutated HCC shows immune suppression^7,16^, the molecular mechanism is not fully clarified in HCC^17^.

In this study, we established a novel model of intrinsically active β-catenin signaling by CRISPR/Cas9-mediated exon skipping in human and mouse HCC cells, and investigated how tumor β-catenin signaling evades the immune system in HCC.

## Results

### Exon 3 skipping of β-catenin by multiplex CRISPR/Cas9-based genome engineering system

**Figure 1:**
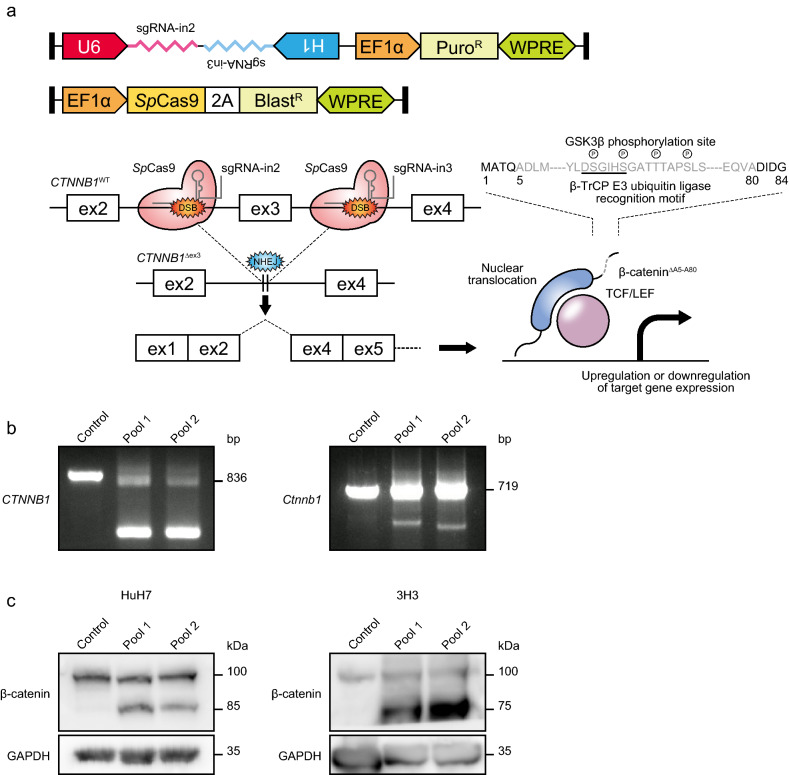
CRISPR/Cas9-mediated exon 3 skipping of β-catenin. (**a**) Schematic representation of exon 3 skipping of β-catenin (*CTNNB1*). (**b**,**c**) PCR and immunoblot analysis of β-catenin in human and mouse HCC cell lines, HuH7 and 3H3. The expected PCR product sizes of wild-type and exon 3-skipping β-catenin are 836 bp, 294 bp (pool 1) and 294 bp (pool 2) in humans and 719 bp, 307 bp (pool 1) and 280 bp (pool 2) in mice, respectively. The molecular weights of β-catenin^wild-type^ and β-catenin^ΔA5–A80^ are 85 kDa and 77 kDa in both humans and in mice. GAPDH was used as a loading control. Full-length gels and blots are presented in Supplementary Figure X.

We newly developed a highly efficient multiplex CRISPR/Cas9-based genome engineering system for exon skipping by modifying the lentiGuide-Puro plasmid (Nat Methods), originally provided from Feng Zhang’s laboratory^18^. We first designed single guide RNAs (sgRNAs) targeting intron 2 (sgRNA-in2) and intron 3 (sgRNA-in3) of human *CTNNB1* gene by using the GPP sgRNA Designer web tool, and constructed *U6*-driven sgRNA-in2 and *H1*-driven sgRNA-in3 expression plasmids, respectively. After confirming the mutation efficiency of the two sgRNAs, we next generated a lentivirus vector for simultaneously expressing them (Fig. 1a), and infected it into the HuH7 cells constitutively expressing *Streptococcus pyogenes* Cas9 nuclease (*Sp*Cas9). As expected, *CTNNB1*^Δex3^ alleles were amplified by PCR of genomic DNA in the genetically engineered HuH7 cells (HuH7-CTNNB1^Δex3^), and β-catenin^ΔA5–A80^ proteins were detected in the pools of the HuH7-CTNNB1^Δex3^ cells (Fig. 1b,c). Similarly to the human HCC cells, mouse HCC cells expressing active form β-catenin were derived from the 3H3 cell line, which was a *Hras*^*Q61L*^-mutated mouse HCC cell line isolated from the C57BL6/J MC4R-KO mouse model^19^, and then termed as 3H3-Ctnnb1^Δex3^. *Ctnnb1*^Δex3^ alleles and β-catenin^ΔA5–A80^ proteins were also identified at the DNA and protein levels in the 3H3-Ctnnb1^Δex3^ pools (Fig. 1b,c). Thus, our multiplex CRISPR/Cas9-based genome engineering system could work efficiently for exon skipping.

### Wnt/β-catenin signaling activation in HCC cells with exon 3 skipping of β-catenin

**Figure 2:**
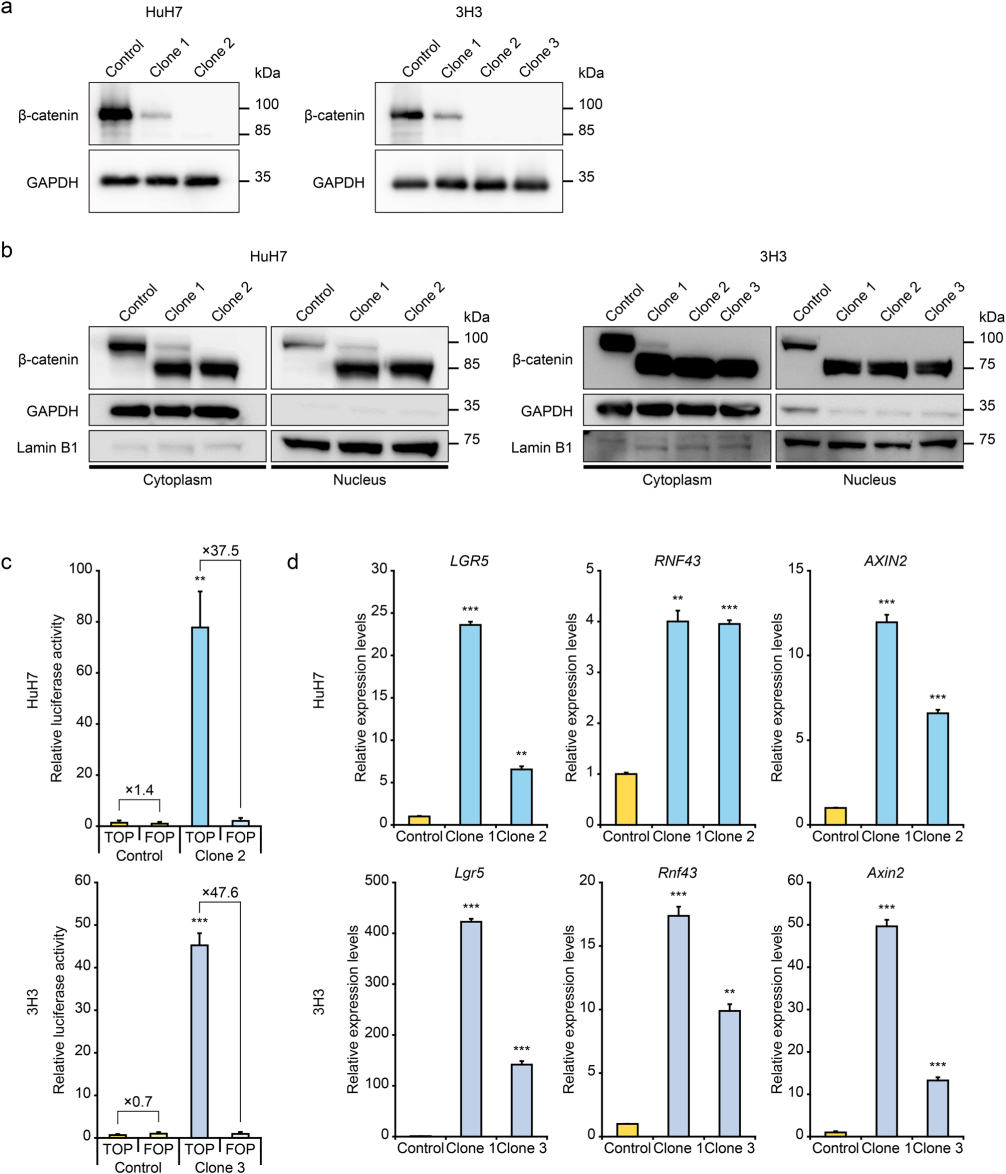
Activation of the Wnt/β-catenin signaling pathway in HCC cells harboring exon 3 skipping β-catenin. (**a**) Immunoblot analysis of β-catenin by using an antibody targeting amino acids 29–49 of β-catenin. Full-length blots are presented in Supplementary Figure X. (**b**) Immunoblot analysis of cytoplasmic and nuclear protein fractions. GAPDH and lamin B1 were used as controls of the cytoplasmic and nuclear protein fractions, respectively. Full-length blots are presented in Supplementary Figure X. (**c**) TOPFlash dual luciferase assay. Luciferase activity of control cells transfected with the FOPFlash vector was used as a control. (**d**) Quantitative RT-PCR analysis of downstream genes of the Wnt/β-catenin signaling pathway. Error bars are the mean ± SD. *P*-values were calculated by Welch's *t* test. **P < 0.01; ***P < 0.001.

Subclones were established from the HuH7-CTNNB1^Δex3^ and 3H3-Ctnnb1^Δex3^ cell pools, and western blotting analysis by using an antibody targeting amino acids 29–49 of β-catenin showed heterozygous (Clone 1 of HuH7-CTNNB1^Δex3^; Clone 1 of 3H3-Ctnnb1^Δex3^) and homozygous (Clone 2 of HuH7-CTNNB1^Δex3^; Clone 2 and Clone 3 of 3H3-Ctnnb1^Δex3^) deletion of exon 3, encoding amino acids 5–80 (Fig. 2a). Nuclear translocation of β-catenin^ΔA5–A80^ proteins was enhanced in the HuH7-CTNNB1^Δex3^ and 3H3-Ctnnb1^Δex3^ subclones (Fig. 2b). The TOPFlash assay demonstrated approximately 40-fold increased activation of the Wnt/β-catenin signaling pathway in the human and mouse *CTNNB1*^Δex3^ cells (Fig. 2c). To determine major downstream genes of the Wnt/β-catenin signaling pathway in HCC, we compared gene expression profiles between human HCC samples with and without mutations in exon 3 of *CTNNB1* by using public genome and transcriptome data sets provided from the Cancer Genome Atlas Research Network (TCGA) as shown in Supplementary Table 1. The differentially expressed genes included key components of the Wnt/β-catenin signal transduction, such as *LGR5*, *RNF43*, *ZNRF3*, *AXIN2* and *TCF7*, implying positive and negative feedback loops. We examined the mRNA expression levels of them, and significant upregulation of *LGR5*, *RNF43* and *AXIN2* indicated activation of the β-catenin signaling in the HuH7-CTNNB1^Δex3^ and 3H3-Ctnnb1^Δex3^ subclones (Fig. 2d). Overexpression of glutamine synthetase, encoded by *GLUL*, is a potential biomarker for β-catenin signaling activation. Immunoblot analysis identified upregulation of glutamine synthetase in the HuH7-CTNNB1^Δex3^ and 3H3-Ctnnb1^Δex3^ cells (Supplementary Fig. 1). WST-8 analysis showed higher proliferative activity of the HuH7 and 3H3 cells with β-catenin signaling activation (Supplementary Fig. 2). Taken together, CRISPR/Cas9-mediated exon 3 skipping of β-catenin could molecularly and biologically mimic the β-catenin signaling activation in human and mouse HCC cells.

### Downregulation of immune-related gene sets by exon 3 skipping of β-catenin in HCC

**Figure 3:**
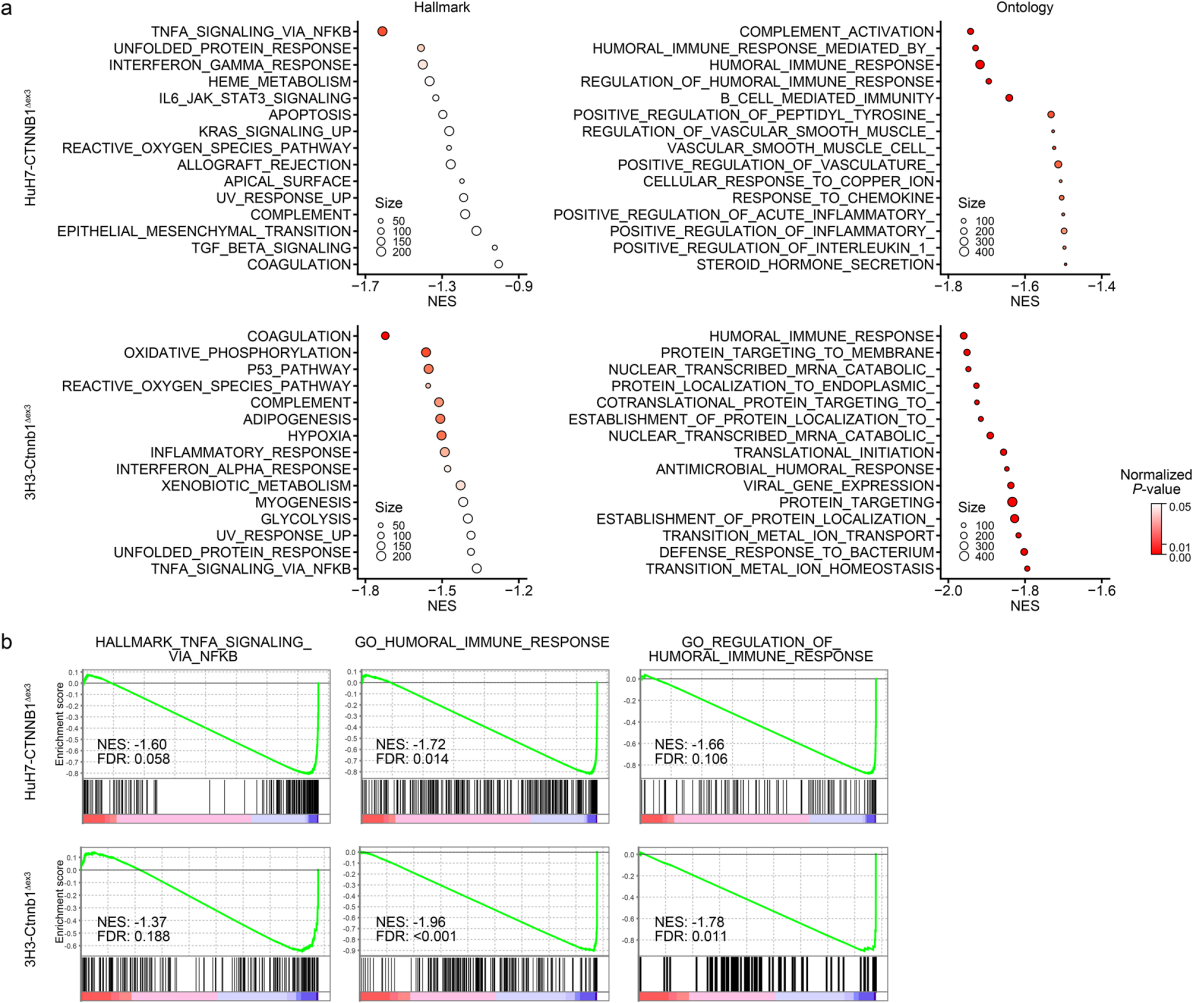
Alteration of signaling pathways by exon 3 skipping of β-catenin in HCC. (**a**) Bubble plots of gene sets negatively enriched in the HuH7-CTNNB1^Δex3^ and 3H3-Ctnnb1^Δex3^ cells. (**b**) Enrichment plots of gene sets commonly associated with the HuH7-CTNNB1^Δex3^ and 3H3-Ctnnb1^Δex3^ cells. Hallmark (H: hallmark) and ontology (C5.BP: gene ontology biological process) gene sets were obtained from the MSigDB. Normalized enrichment score (NES), normalized *P*-value and false discovery rate (FDR) were calculated by the GSEA application.

We performed RNA-seq analysis of the HuH7-CTNNB1^Δex3^ and 3H3-Ctnnb1^Δex3^ cells, and identified *LGR5*, *RNF43*, *AXIN2* and *TMPRSS2* as commonly upregulated genes (log2 fold-change > 1.5 and *P*-value < 10^–10^), which was consistent with the results of quantitative RT-PCR analysis. Gene set enrichment analysis (GSEA) of the HuH7-CTNNB1^Δex3^ and 3H3-Ctnnb1^Δex3^ cells revealed the close relationship between activation of the β-catenin signaling and downregulation of immune-associated gene sets (Fig. 3a). The HALLMARK TNFA SIGNALING VIA NFKB (M5890), GO HUMORAL IMMUNE RESPONSE (M13774) and GO REGULATION OF HUMORAL IMMUNE RESPONSE (M14968) gene sets were negatively enriched in both of the human and mouse HCC cells (Fig. 3b). These findings suggested that β-catenin signaling activation could contribute to immune evasion, and were consistent with previous studies of clinical specimens^7,13,16^.

### Downregulation of cytokine genes in HCC with β-catenin signaling activation

**Figure 4:**
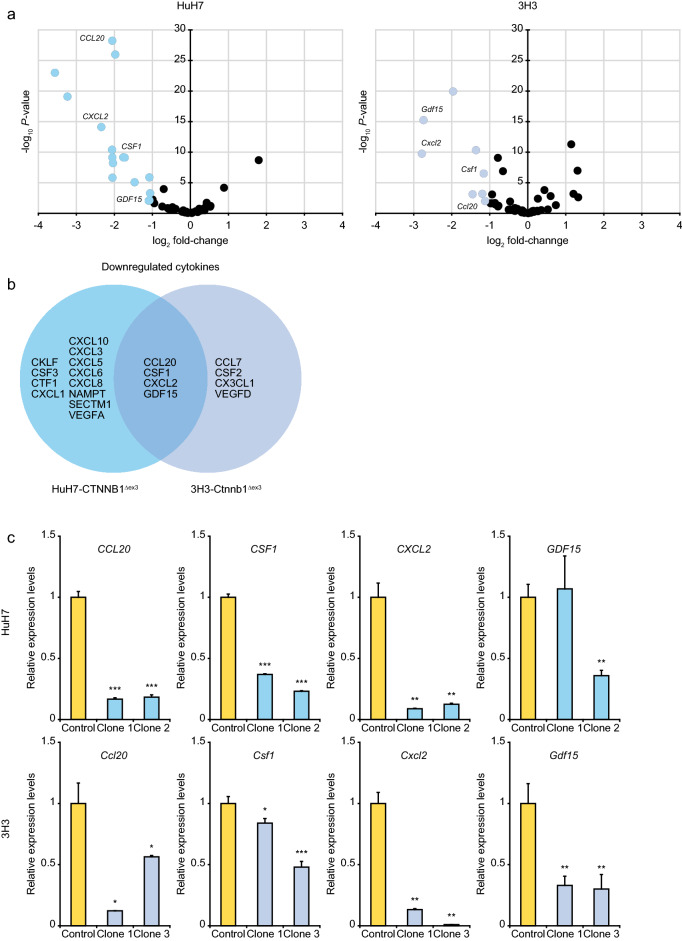
Downregulation of cytokines in human and mouse HCC cells with exon 3 skipping of β-catenin. (**a**) Volcano plots of differentially expressed cytokine genes between the HuH7 and 3H3 cells with and without β-catenin signaling activation. (**b**) Venn diagram of cytokine genes downregulated in the HuH7-CTNNB1^Δex3^ and 3H3-Ctnnb1^Δex3^ cells. Twenty and sixteen genes were extracted from 114 genes registered in the CYTOKINE ACTIVITY gene set (fold-change < 0.5 and *P*-value < 0.01). (**c**) Quantitative PCR analysis of four candidate cytokine genes downregulated by β-catenin signaling activation. Error bars are the mean ± SD. *P*-values were calculated by Welch's *t* test. *P < 0.05; **P < 0.01; ***P < 0.001.

**Figure 5:**
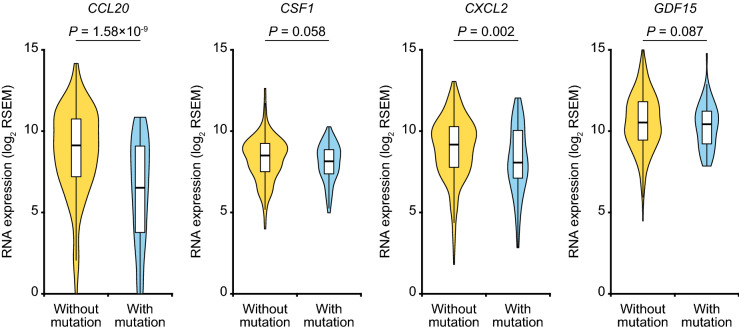
Downregulation of cytokines in human HCC samples with *CTNNB1* hotspot mutations. Boxes in violin plots represent the interquartile range (range from the 25th to the 75th percentile), and horizontal lines show the median values. *P*-values were calculated by Mann–Whitney *U* test.

Among 114 cytokines registered in the CYTOKINE ACTIVITY gene set (M14581), 16 and 8 cytokines were remarkably downregulated in human and mouse *CTNNB1*^Δex3^ HCC cells, respectively (Fig. 4a and Supplementary Table 2a,b), and *CCL20*, *CSF1*, *CXCL2* and *GDF15* were commonly suppressed at the mRNA level (Fig. 4b), which was confirmed by quantitative RT-PCR analysis (Fig. 4c). Knockdown of β-catenin recovered the expression levels of *CCL20*, *CSF1* and *GDF15* in the HuH7-CTNNB1^Δex3^ cells, whereas recovering the expression level of *Ccl20* in the 3H3-Ctnnb1^Δex3^ cells (Supplementary Fig. 3). We next compared the expression levels of the four cytokine genes between HCC samples with and without mutations in exon 3 of *CTNNB1* by using the TCGA data sets (Fig. 5), and identified significant downregulation of *CCL20* (fold-change: 0.164; *P*-value: 1.58 × 10^–9^) and *CXCL2* (fold-change: 0.467; *P*-value: 0.002).

### Immune evasion of mouse HCC cells with exon 3 skipping of β-catenin

**Figure 6:**
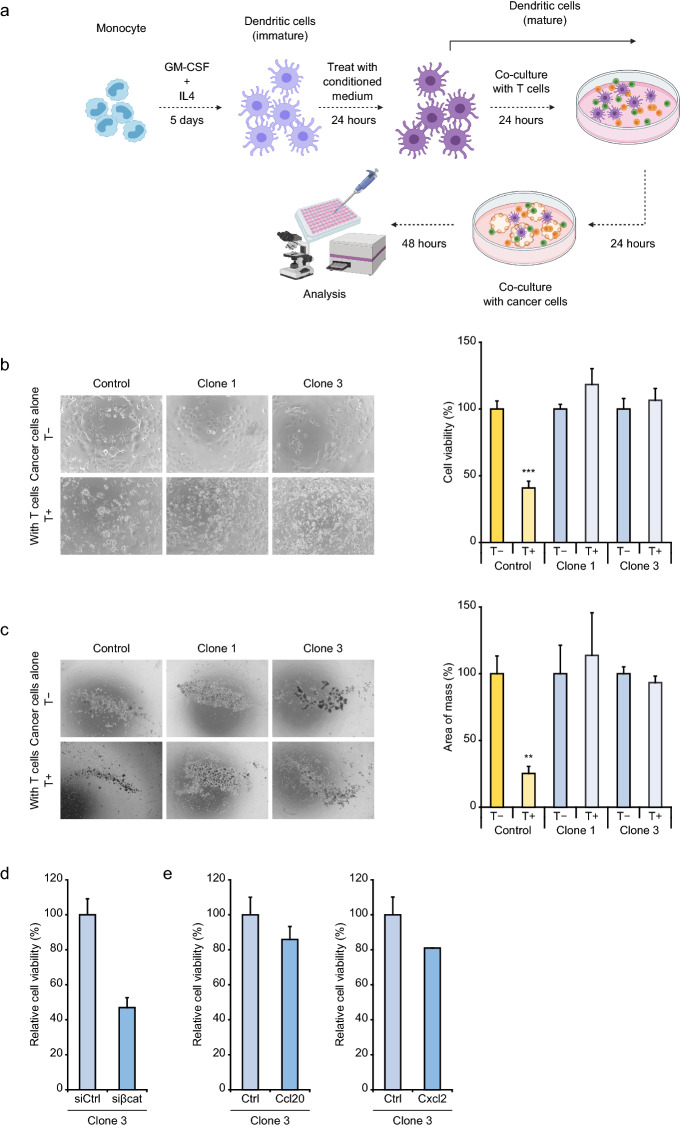
Immune evasion of mouse HCC cells with β-catenin signaling activation. (**a**) Schematic representation of in vitro T cell killing assays. (**b**,**c**) Two- and three-dimensional T cell killing assays of the 3H3-Ctnnb1^Δex3^ cells. The left and right panels show representative phase-contrast images and cell viability data, respectively. Error bars are the mean ± SD. *P*-values were calculated by Welch's *t* test. **P < 0.01; ***P < 0.001. (**d**) and (**e**) Three-dimensional T cell killing assays of the β-catenin-knockdown and cytokine-expressing 3H3-Ctnnb1^Δex3^ cells, respectively. Error bars are the mean ± SD.

For evaluating inhibitory effects of the β-catenin signaling on immune surveillance, we investigated T cell killing of the 3H3-Ctnnb1^Δex3^ and control cells (3H3-Ctrl) as illustrated in Fig. 6a. Monocytes and T lymphocytes were obtained from C57BL6/J mice, and activated by conditioned media of each cell lines. By co-culture with immune cells, the number of the 3H3-Ctrl cells was notably decreased by more than 50%, while the number of the 3H3-Ctnnb1^Δex3^ cells was not changed (Fig. 6b). Three dimensional co-culture system also showed the similar results (Fig. 6c). Knockdown of β-catenin in the 3H3-Ctnnb1^Δex3^ cells strikingly restored the T cell killing effect (Fig. 6d). For examining the role of cytokines downregulated by β-catenin signaling activation, we generated the 3H3-Ctnnb1^Δex3^ cells ectopically expressing mouse *Ccl20* and *Cxcl2*. Both *Ccl20* and *Cxcl2* overexpression in the 3H3-Ctnnb1^Δex3^ cells elicited 14.0% and 19.0% reduction of cancer cell viability compared with control cells, respectively, indicating that overexpression of the two candidate cytokines could show a rescue of T cell killing (Fig. 6e).

**Figure 7:**
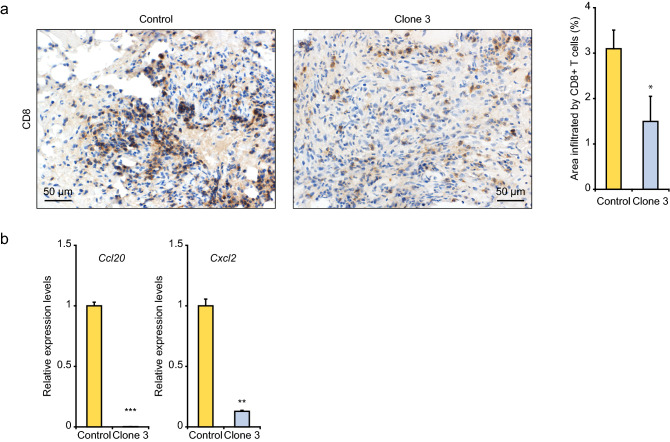
Immune evasion of mouse HCC tissues with β-catenin signaling activation. (**a**) CD8+ T cells in grafted tumor tissues. The left and right panels show representative immunohistochemical images and T cell infiltration data, respectively. (**b**) Quantitative PCR analysis of cytokine genes in grafted tumor tissues. Error bars are the mean ± SD. *P*-values were calculated by Welch's *t* test. *P < 0.05.

We next evaluated tumorigenic activity of the 3H3-Ctnnb1^Δex3^ and 3H3-Ctrl cells by subcutaneously injection into C57BL6/J mice. Glutamine synthetase expression, which is a surrogate marker for β-catenin signaling activation in HCC, was enhanced in the grafted tumors of the 3H3-Ctnnb1^Δex3^ cells. There was no difference of Ki-67 staining between the tumor specimens of the 3H3-Ctrl and 3H3-Ctnnb1^Δex3^ cells (Supplementary Fig. 4). Remarkably, the tumor size of the 3H3-Ctnnb1^Δex3^ cells was larger than that of the 3H3-Ctrl cells (Supplementary Fig. 5), and immunohistochemical analysis revealed decreased CD8+ T cell infiltration in the tumor tissue of the 3H3-Ctnnb1^Δex3^ cells (Fig. 7a). The tumors of the 3H3-Ctnnb1^Δex3^ cells showed a dramatic decrease of *Ccl20* and *Cxcl2* expression (Fig. 7b).

## Discussion

Although previous studies have examined the relationship between the Wnt/β-catenin signaling pathway and immune surveillance, they are artificial due to overexpression of mutated β-catenin, such as β-catenin^S37F^ driven by SV40 promoter^14^, β-catenin^S33A;S37A;T41A;S45A^ driven by tyrosinase promoter^15^, and β-catenin^ΔN90^ driven by EF1α promoter^17^. To overcome this limitation, we first tried to knock in a mutated sequence of *CTNNB1* exon 3 to human HCC cells with the help of the CRISPR/Cas9 system by using single strand DNA or plasmid donor, but it is difficult to obtain *CTNNB1*-mutated subclones because homology-directed repair is less dominant than non-homologous end joining (NHEJ) during double-strand break repair. We then mimicked activation of the β-catenin signaling by skipping exon 3, that is, joining the ends of intron 2 and intron 3 simultaneously cleaved by the CRISPR/Cas9 system. For this purpose, we established the novel multiplex CRISPR/Cas9-mediated genome engineering system of lentiCas9-Blast and improved lentiGuide-Puro plasmids, although Kabadi et al*.* have already produced a lentivirus vector containing a Cas9 transcription cassette and multiple sgRNA transcription cassettes^20^. This is because a Golden Gate cloning method is complicated compared with a conventional cloning method, and because two-vector system is superior to all-in-one vector system in functional viral titer^18^. Thus, our vector system enabled exon skipping with ease and efficiency, and could expand to other models, such as *EGFR*^Δex19^ and *ERBB2*^Δex16^ for activation of the EGFR signaling pathway, and *POLD1*^Δex10^ and *POLE*^Δex9^ for attenuation of exonuclease activity.

Immune checkpoint inhibitors (ICIs) including anti-PD-1 and anti-PD-L1 antibodies have provided a revolutionary approach to cancer therapy, and clinical trials of ICIs for various types of cancer are now ongoing and successful. In HCC, two anti-PD-1 antibodies nivolumab and pembrolizumab prolonged patient survival in phase II trials, however both monotherapies failed in phase III trials unfortunately. Harding et al*.* have revealed that *CTNNB1*-mutated HCC is more accumulated in the ICI-resistant group than in the ICI-sensitive group^21^, which is consistent with the important finding that *CTNNB1* mutation is enriched in non-T cell-inflamed tumors insusceptible to ICI therapy^13^. Since the mutation rate of *CTNNB1* gene is relatively higher in HCC than in other types of cancer (Supplementary Fig. 6), clinical trials of ICIs should be conducted or subanalyzed for HCC with wild-type and mutated β-catenin separately. As described above, it is possible that the β-catenin signaling regulates not immune checkpoint molecules but cytokines for control of tumor immune microenvironment, such as upregulation of IL-10^14^ and downregulation of CCL4^15^. Ruiz de Galarreta et al*.* demonstrated that antigen-expressing *MYC*;*Trp53*^−/−^ HCC evaded the immune system by decreasing CCL5 expression through activation of the β-catenin signaling pathway, and that CCL5 overexpression restored immunosurveillance in antigen-expressing *MYC*;*CTNNB1*^*ΔN90*^ HCC^17^. In contrast, this study demonstrated that endogenous active form β-catenin downregulated immune-associated signaling pathways in both human and mouse HCC by bioinformatic analysis, and that tumor-intrinsic β-catenin activation suppressed T cell cytotoxicity through cytokine secretion by in vitro assays.

By comparing the present and previous studies^17^, CCL20 was commonly downregulated in HCC with β-catenin signaling activation, although there was no difference of CCL5 expression between human and mouse HCC cells with and without exon 3 skipping of β-catenin in this study. CCL20, alternatively named liver and activation-regulated chemokine (LARC), was originally discovered in the liver and strongly expressed in mononuclear cells near necrosis in the chronically inflamed liver and HCC. CCR6 is the selective receptor for CCL20, and the CCR6-CCL20 axis contributes to the recruitment of immature DC to the antigen entry site and the arrest of T lymphocyte on the endothelium in the early phase of immune response^22^. The chemokine receptor CXCR2 and its ligands CXCL1, CXCL2, CXCL3, CXCL5 and CXCL8 play critical roles in the chemoattraction of neutrophils towards tumor tissues. Similarly to tumor-associated macrophages, tumor-associated neutrophils can be polarized into either an antitumoral (N1) or a protumoral (N2) phenotype; the N1 phenotype is induced by TGF-β blockade, and expresses immunoactivating cytokines and chemokines for killing cancer cells^23^. Thus, the β-catenin signaling pathway might suppress immune response through decrease of cytokine levels.

We hypothesized two molecular mechanisms underlying downregulation of cytokines by β-catenin signaling activation. One is that active form β-catenin might indirectly suppress cytokine expression by controlling other transcription factors. Spranger et al*.* have previously reported that the Wnt/β-catenin signaling induces expression of a transcription repressor ATF3, and that ATF3 inhibits CCL4 expression in melanoma cells^15^, but the expression levels of transcription factors including ATF3 were not altered in human and mouse HCC cells with β-catenin signaling activation. The other is that β-catenin might change the epigenetic status of cytokine genes. Large-scale methylome data of HCC provided from the Cancer Genome Atlas Research Network clarified that downstream genes of the Wnt/β-catenin signaling pathway, such as *LGR5*, *RNF43* and *AXIN2*, were demethylated at the promoter regions in *CTNNB1*-mutated HCC samples (Supplementary Table 3). However, the methylation levels of the promoter regions of *CCL20* or *CXCL2* were not different between HCC samples with and without *CTNNB1* mutations, implying that other epigenetic systems such as histone modification might be involved in downregulation of cytokine genes^25^. In addition, since cross-regulation between the Wnt/β-catenin and NFκB signaling pathways has recently been identified in various types of cancer^26^, the similar molecular mechanism might also function in HCC cells.

In conclusion, this study enabled intrinsic β-catenin signaling activation by developing the highly efficient CRISPR/Cas9-based exon skipping system, and showed that it could contribute to immune evasion by suppressing immunoactivating cytokines including CCL20 and CXCL2. The *CTNNB1*-mutated HCC subtype accounts for approximately 30% of all cases (Supplementary Fig. 6), but is refractory to ICI therapy. Since clinical trials evaluating recombinant cytokines as immunostimulants in cancer patients have recently been launched^24^, transarterial infusion of the candidate immunoactivating cytokines could also be effective to the subtype.

## Methods

### Ethics statement

The study was carried out in compliance with the ARRIVE guidelines. All methods were performed in accordance with relevant guidelines and regulations. All experimental protocols were approved by Institutional Review Board (G2018-132C5, Medical Research Ethics Committee for Genetic Research of Tokyo Medical and Dental University; A2019-263C2, Institutional Animal Care and Use Committee of Tokyo Medical and Dental University).

### Cell culture

Human HCC cell line HuH7 was purchased from the American Type Culture Collection (Manassas, VA). Mouse cell line 3H3 was derived from HCC tumor grown in a C57BL/6J MC4R-KO mouse fed with high fat diet^19^. We examined mutations of *Ctnnb1*, *Trp53*, *Braf* and *Hras*, which are frequently observed in human HCC and carcinogen-induced mouse HCC, and only detected the *Hras*^Q61L^ mutation in the 3H3 cells. They were cultured in RPMI-1640 and DMEM (Wako, Osaka, Japan) medium containing 10% fetal bovine serum (FBS), and 1% penicillin, streptomycin and amphotericin B (Wako), maintained in a humidified incubator at 37 °C in 5% CO_2_, and harvested with 0.05% trypsin-0.03% EDTA (Wako).

### Exon 3 skipping of β-catenin by multiplex CRISPR/Cas9-based genome engineering system

To generate the backbone plasmid for the CRISPR/Cas9 system, the lentiGuide-Puro (Addgene #52963) was modified by inserting a *Kpn*I site in front of the *U6* promoter and replacing the *Hind*III site behind the sgRNA scaffold with an *Eco*RI site, named as LG-U6. The LG-H1 plasmid was also produced by replacing the *U6* promoter with the *H1* promoter in the LG-U6 plasmid. The LG-U6 and LG-H1 plasmid for expressing sgRNAs targeting intron 2 and intron 3 of β-catenin (sgRNA-in2 and sgRNA-in3) were constructed following the manufacture’s manual (Supplementary Table 4). The H1-sgRNA-in3 sequence was tandemly cloned into the *Eco*RI site of the LG-U6-sgRNA-in2 plasmid (Fig. 1a). The HuH7 and 3H3 cells were sequentially infected with the lentiviral vectors for constitutively expressing *Sp*Cas9 (lentiCas9-Blast; Addgene #52962) and simultaneously expressing sgRNA-in2 and sgRNA-in3, and then treated with 10 μg/mL blasticidin and 10 μg/mL puromycin, respectively. The subclones with β-catenin alleles lacking exon 3 were isolated by limiting dilution.

### DNA extraction and PCR analysis

Cell pellets were suspended in TNE Buffer (10 mM Tris–HCl, pH 8.0; 150 mM NaCl; 2 mM EDTA; 0.5% SDS) with 1% proteinase K (TaKaRa Bio, Shiga, Japan) at 55 °C overnight. Genomic DNA was obtained from cells by phenol–chloroform extraction. The primer sets and amplification conditions for PCR are listed in Supplementary Table 5.

### RNA extraction

Total RNA was extracted from cells by using RNeasy Plus Mini Kit (QIAGEN, Germantown, MD). Contaminating DNA was removed by digestion with RNase-Free DNase Set (QIAGEN).

### Quantitative RT-PCR analysis

For single-stranded complementary DNA synthesis, 1 μg of total RNA was reverse-transcribed by SuperScript III Reverse Transcriptase (Thermo Fisher Scientific, Waltham, MA). Quantitative RT-PCR analysis was performed by using TB Green Premix Ex Taq II (TaKaRa Bio) with StepOne real-time PCR system (Thermo Fisher Scientific) according to the manufacturer’s instructions, and the ΔΔCt method was used for quantification. GAPDH was used as an internal control. The primer sets and amplification conditions for PCR are listed in Supplementary Table 6.

### RNA sequencing analysis

Sequencing libraries were prepared from total RNA with the TruSeq Standard mRNA Library Kit (Illumina, San Diego, CA), and RNA sequencing was run on an Illumina NovaSeq 6000. Sequence reads were aligned to the human and mouse reference genome (GRCh38 and GRCm38) by STAR (2.7.0d), and transcript quantification was performed by RSEM (1.3.1). Differentially expressed genes were determined by using DESeq2 (1.14.1).

### Western blotting

After whole cell lysates were collected by using ice-cold RIPA buffer (Thermo Fisher Scientific), 30 μg of protein from each sample was subjected to electrophoresis through 10% sodium dodecyl sulfate–polyacrylamide gels and transferred onto Immobilon polyvinyldifluoride membranes (Millipore, Bedford, MA). The membrane was blocked with 5% skimmed milk or bovine serum albumin for an hour at room temperature, adequately cut, and then incubated overnight at 4 °C with primary antibodies as follows;β-catenin (D10A8, 1:1000, for detection of the C-terminal region), GAPDH (14C10, 1:1000) and lamin B1 (D6V6H, 1:1000), all of which were purchased from Cell Signaling Technology (Danvers, MA); β-catenin (06-734, 1:1000, for detection of amino acids 29-49) from Millipore-Sigma (St Louis, MO); glutamine synthetase (ab64613, 1:500) from Abcam (Cambridge, UK). Secondary antibodies were added, and signals were detected by using Clarity Western ECL Substrate (Bio-Rad, Hercules, CA) with LAS-3000 (Fujifilm, Tokyo, Japan). The original image data of blots were shown in Supplementary Figure X, although full-length images with membrane edges visible were not fully provided.

### Subcellular fractionation analysis

Cytoplasmic and nuclear proteins were separately extracted by NE-PER Nuclear and Cytoplasmic Extraction Reagents (Thermo Fisher Scientific) according to the manufacturer’s instructions, and then Western blotting analysis was performed. GAPDH and lamin B1 were used for detecting cytoplasmic and nuclear fractionated protein, respectively.

### TOPFlash dual luciferase reporter assay

The M50 Super 8× TOPFlash plasmid (#12456) and the M51 Super 8× FOPFlash plasmid (#12457) from Addgene (Watertown, MA). The HuH7 and 3H3 cells were seeded at a density of 1 × 10^5^ and 3 × 10^4^ cells per well in 24-well plates, respectively, and transiently transfected with 400 ng TOPFlash or FOPFlash plasmids and 100 ng pNL1.1.TK plasmid (Promega, Madison, WI) as an internal control by using PEI MAX (Polysciences, Warrington, PA). Two days after transfection, the relative light unit (RLU) was measured by using Nano-Glo Dual Luciferase Reporter Assay System (Promega) with FLUOstar OPTIMA-6 microplate reader (BMG Labtech, Durham, NC) following the manufacturer’s instructions. Luciferase activity was calculated as the ratio of firefly RLU (TOPFlash or FOPFlash) to NanoLuc RLU (pNL1.1.TK).

### Cell proliferation analysis

Cells were seeded at a density of 5 × 10^3^ cells per well in 96-well plates, and incubated overnight before each assay. The number of cell lines was estimated by using WST-8 in accordance with the manufacturer’s instructions. Briefly, 1 h after 100 μL of Cell Counting Kit-8 solution (Dojindo, Kumamoto, Japan) were added to each well, the absorbance was measured on a microplate reader (Bio-Rad Laboratories, Hercules, CA) at 450 nm.

### Knockdown of β-catenin

Control siRNA duplex (siCtrl; MISSION siRNA Universal Negative Control) and β-catenin-targeting siRNA duplex (siβcat; SASI_Hs01_00117960 for human *CTNNB1* and SASI_Mm01_00161710 for mouse *Ctnnb1*) were purchased from Merck KGaA (Darmstadt, Germany), and transfected into cells by using Lipofectamine RNAiMAX Transfection Reagent (Thermo Fisher Scientific).

### Isolation of T cells

Eight-week-old male C57BL/6J mice were euthanized, and spleens were resected and disrupted with a flat plunger tip of a 5 mL syringe. After hemolysis, whole splenocytes were incubated in a nylon wool fiber column to remove B lymphocytes for an hour at 37 °C. T lymphocytes were collected and cultured in RPMI-1640 medium supplemented with 10% FBS, 1% ITS supplement (Thermo Fisher Scientific), 100 U/mL murine IL-2 (Peprotech, Cranbury, NJ) and 10 ng/mL murine IL-7 (Peprotech).

### Immune-cell preparation

Isolation of mouse bone marrow and differentiation of DCs was performed as previously described^27^. Briefly, eight-week-old male C57BL/6J mice were euthanized, and bone marrow was flushed out from femur and tibia by using a 1 mL syringe and a 27G needle. Bone marrow-derived monocytes (BMDMs) were washed, and then cultured in DC differentiation medium as follows; RPMI-1640, 10% FBS, 1% penicillin–streptomycin-amphotericin B, 20 ng/mL murine GM-CSF (Peprotech) and 5 ng/mL murine IL-4 (Peprotech). Six days after preculture, differentiated bone marrow-derived dendritic cells (BMDCs) were further cultured in conditioned medium collected from the 3H3-Ctrl cells or 3H3-CTNNB1^Δex3^ cells for 24 h to stimulate with cancer antigens.

### T cell killing assay

A day after T lymphocytes were co-cultured with BMDCs for priming, cells were plated at 5 × 10^3^ cells per well in a 24-well tissue culture plate (for two-dimensional culture) or ultra-low attachment plate (for sphere culture) with primed immune cells for 48 h. Advanced DMEM/F12 (Thermo Fisher Scientific) with 0.5% B-27 supplement (Thermo Fisher Scientific), 20 ng/mL human EGF (Peprotech) and 1 μg/mL human FGF-basic (Peprotech) was used for sphere formation. To evaluate cytotoxic activity of immune cells in two-dimensional culture, cell viability was estimated by using CellTiter-Glo 2.0 reagent (Promega) with FLUOstar OPTIMA-6 microplate reader (BMG Labtech) according to the manufacturer’s instructions. For sphere culture, cancer cell area was measured by using ImageJ software.

### Tumor seeding

After suspended in 100 μL Matrigel (BD Biosciences, Franklin Lakes, NJ), 1 × 10^6^ cells were subcutaneously injected into C57BL6/J mice. Ten days after transplantation, mice bearing tumors were sacrificed, and the tumors were resected.

### Immunohistochemical analysis

Tissues were fixed overnight in 4% paraformaldehyde, embedded in paraffin, and sectioned (4 μm thick). Sections were immersed in sodium citrate (pH 6.0) buffer for antigen retrieval, and subsequently incubated with primary antibodies against CD8 (D4W2Z, 1:500; Cell Signaling Technology), glutamine synthetase (ab64613, 1:100; Abcam) and Ki-67 (D3B5, 1:200; Cell Signaling Technology) at 4 °C overnight. They were probed with anti-mouse or anti-rabbit IgG antibody labelled with peroxidase Histofine Simple Stain MAX-PO (Nichirei Bioscience, Tokyo, Japan), and visualized with diaminobenzidine. Nuclei were stained with hematoxylin.

### Overexpression of cytokine genes

Entire coding sequence of mouse *Ccl20* and *Cxcl2* were amplified by using PrimeSTAR MAX DNA Polymerase (TaKaRa Bio), and cloned into the *Xho*I and *Not*I sites of the CSII-EF-MCS-IRES-Hygro plasmid^28^. Cells were infected with the lentiviral vectors, and then treated with 500 μg/mL hygromycin.

### Bioinformatic analysis

Gene set enrichment analysis was performed with the MSigDB gene sets. Public genome, methylome and transcriptome data of 373 HCC samples were provided from the Cancer Genome Atlas Research Network, and downloaded from the cBioPortal site. Genome data divided them into 77 and 296 tumors with and without *CTNNB1* hotspot mutations.

## Supplementary Information

Supplementary Information.
