# Supplementary material for: Intrinsic activation of β-catenin signaling by CRISPR/Cas9-mediated exon skipping contributes to immune evasion in hepatocellular carcinoma
Source: Sci Rep. 2021 Aug 24;11:16732. doi: 10.1038/s41598-021-96167-0 (PMC8384852; doi:10.1038/s41598-021-96167-0)
Supplement: Supplementary file 1 — Supplementary Information. [file 41598_2021_96167_MOESM1_ESM.pdf]

Supplementary figure legends

Supplementary Figure 1. Immunoblot analysis of glutamine synthetase in the HuH7-CTNNB1$^{\Delta ex3}$ and 3H3-Ctnnb1$^{\Delta ex3}$ cells. GAPDH was used as a loading control. Full-length blots are presented in Supplementary Figure X.

Supplementary Figure 2. Cell proliferation analysis of the HuH7-CTNNB1$^{\Delta ex3}$ and 3H3-Ctnnb1$^{\Delta ex3}$ cells. Error bars are the mean ± SD. *P*-values were calculated by Welch's *t*-test. **P* < 0.05; ****P* < 0.001.

Supplementary Figure 3. Quantitative PCR analysis of four candidate cytokine genes in the β-catenin-knockdown HuH7-CTNNB1$^{\Delta ex3}$ and 3H3-Ctnnb1$^{\Delta ex3}$ cells. Error bars are the mean ± SD. *P*-values were calculated by Welch's *t*-test. **P* < 0.05; ***P* < 0.01; ****P* < 0.001.

Supplementary Figure 4. Representative immunohistochemical images of glutamine synthetase (**a**) and Ki-67 (**b**) in tumor tissues.

Supplementary Figure 5. Immunohistochemical images of CD8+ T cells in tumor tissues.

Supplementary Figure 6. Bubble plots of *CTNNB1* mutation rate across TCGA cancer types (https://docs.icgc.org/submission/projects/). Red circles represent liver cancer studies.

Supplementary Figure 1

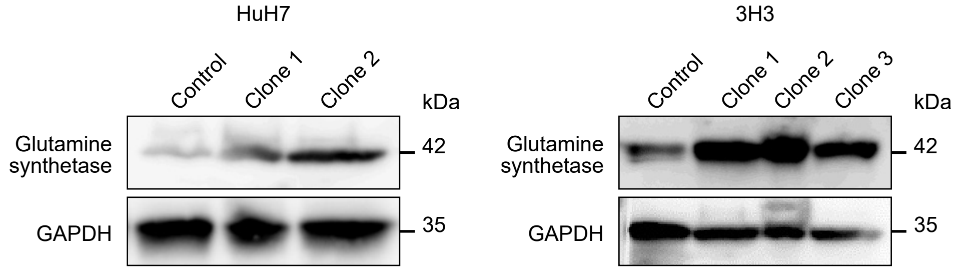

Supplementary Figure 2

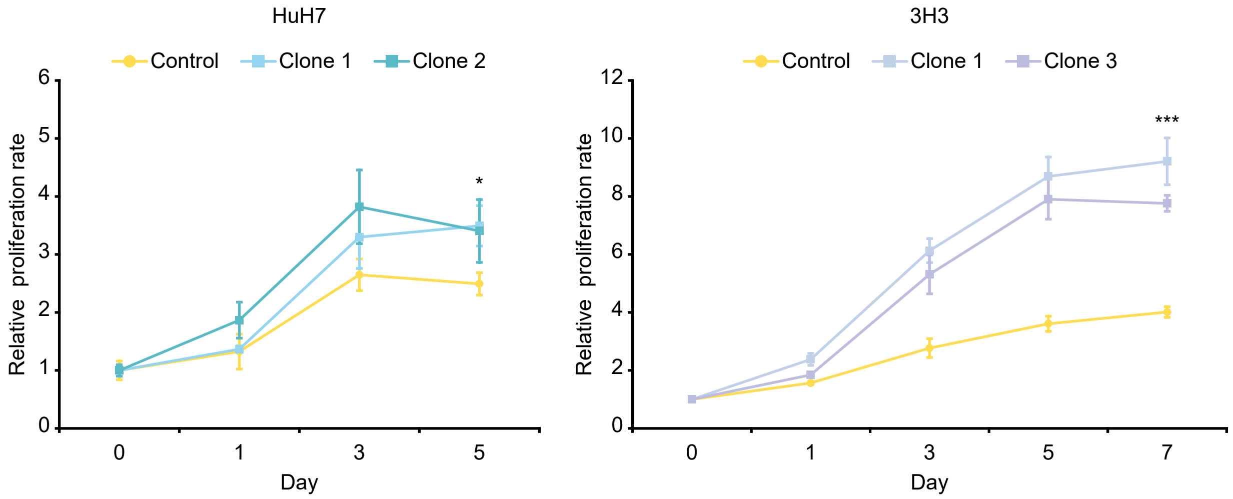

Supplementary Figure 3

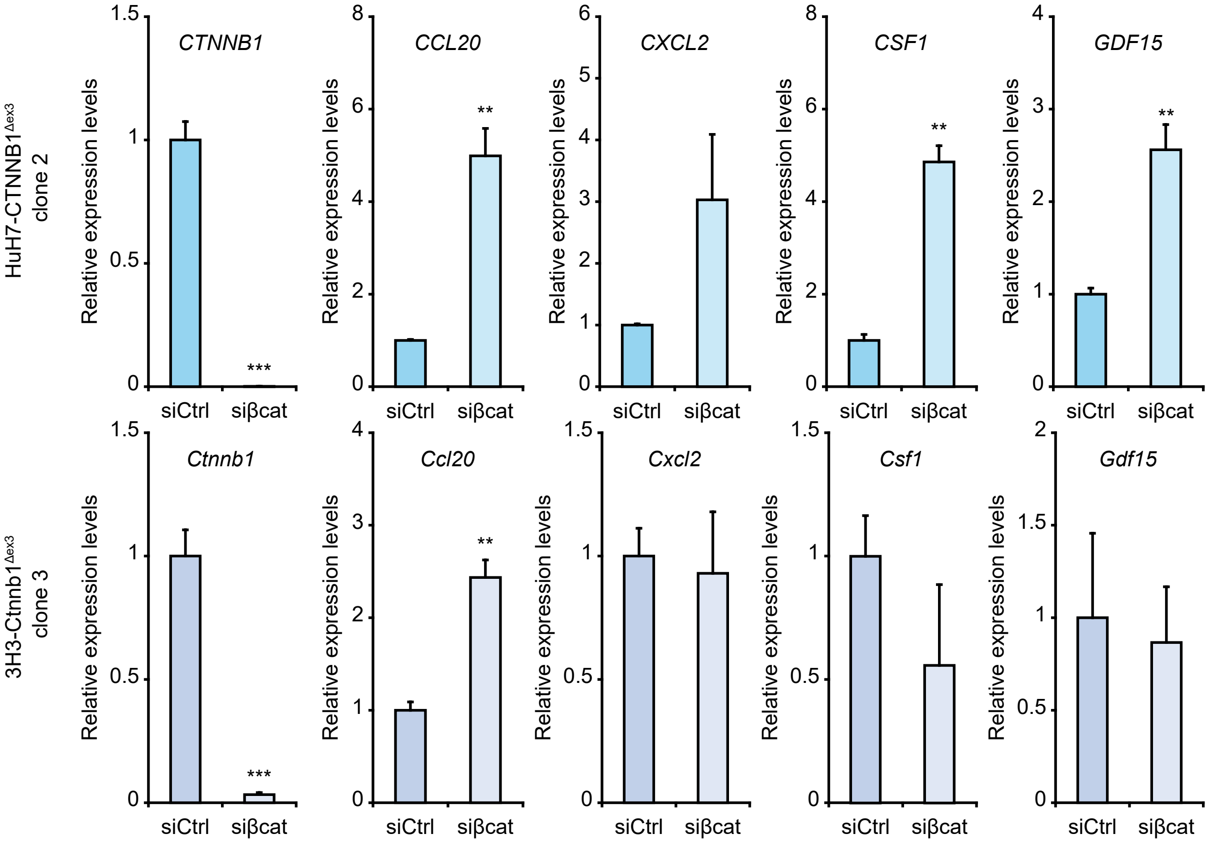

Supplementary Figure 4

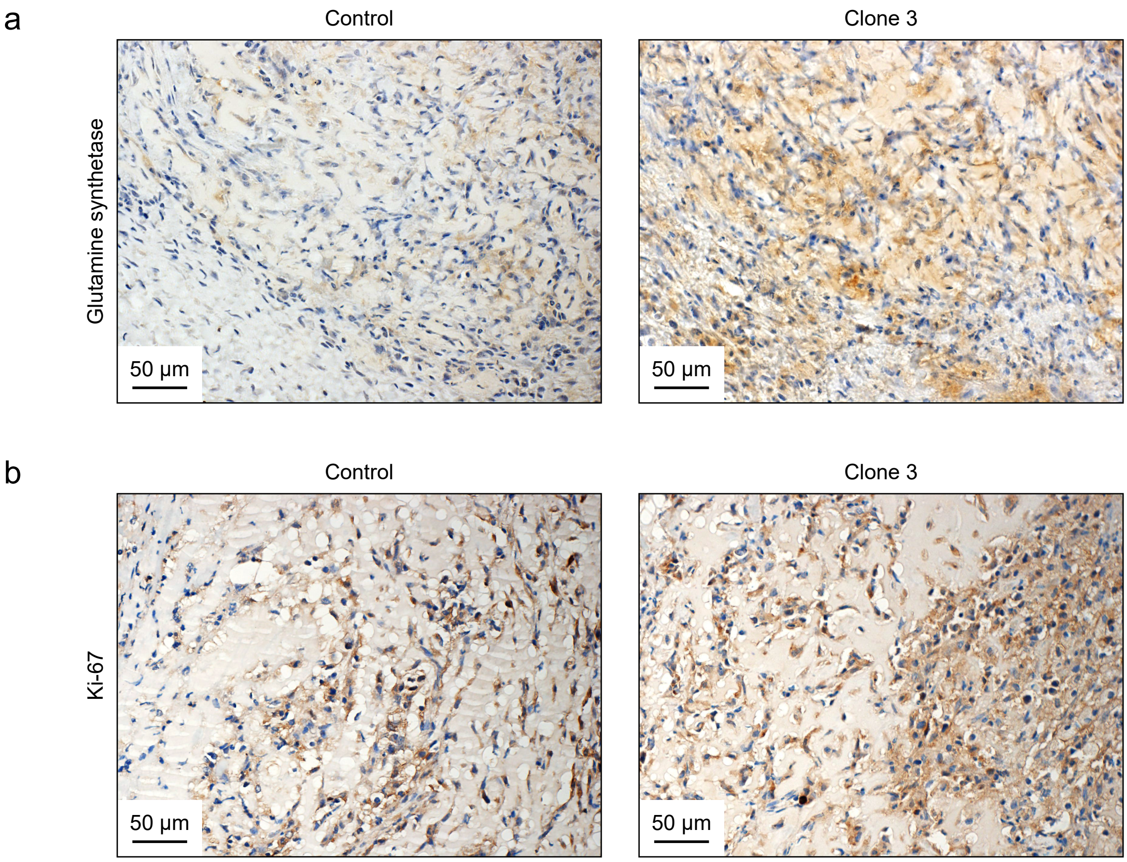

Supplementary Figure 5

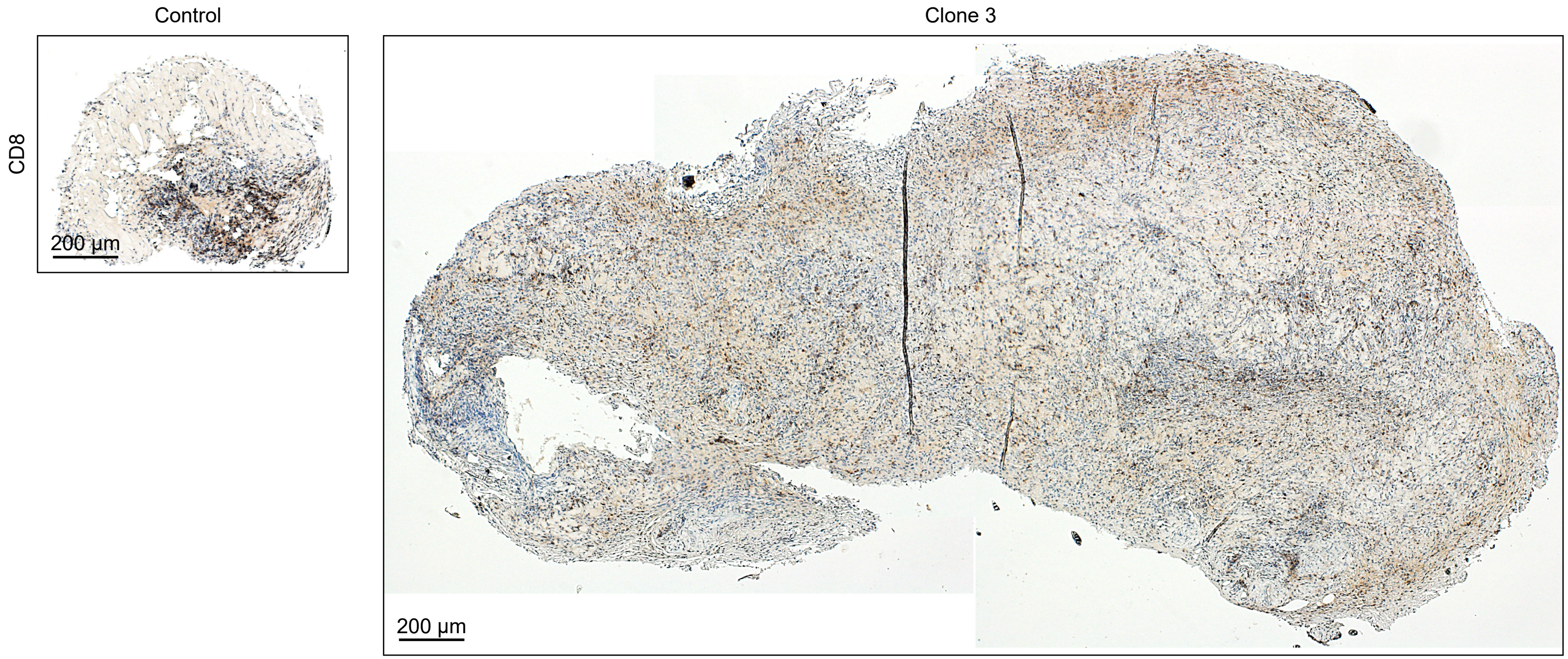

Supplementary Figure 6

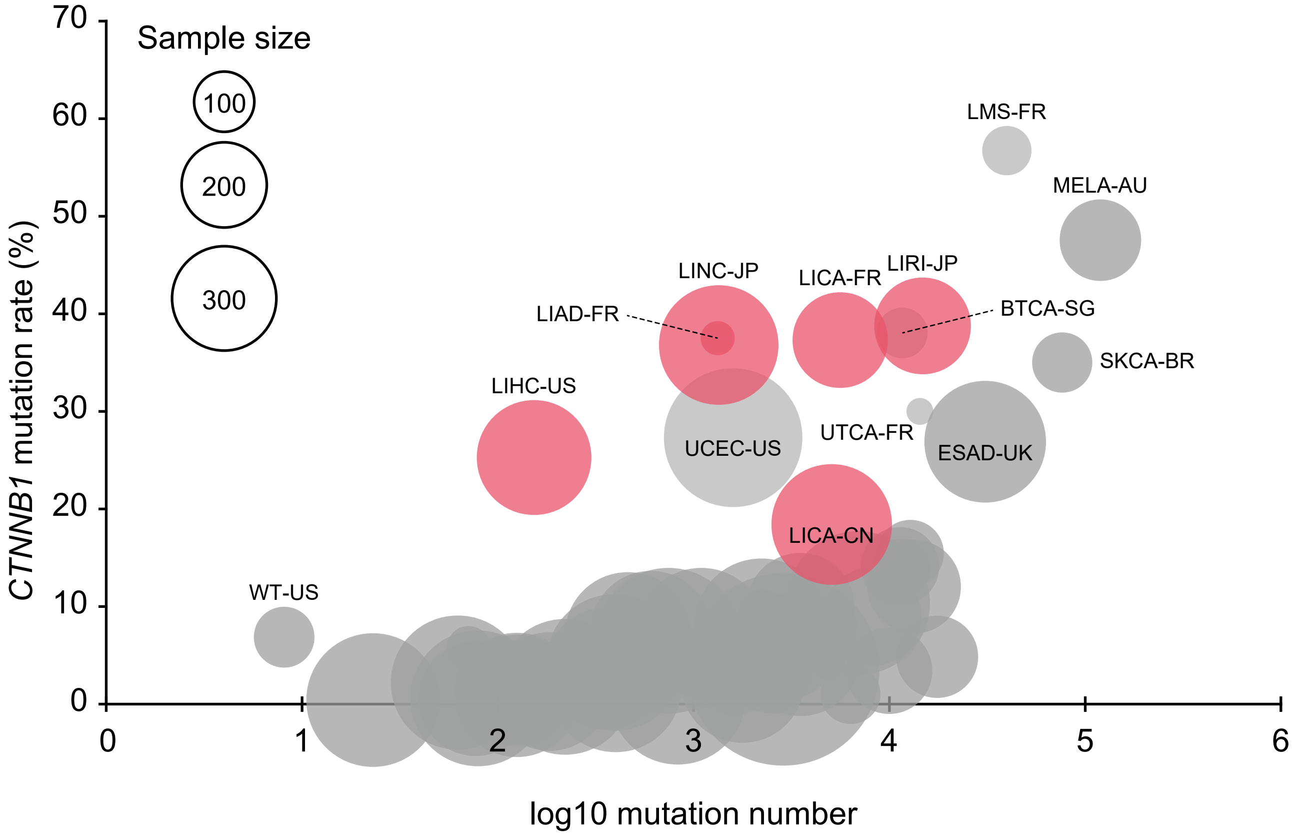

Supplementary Figure X

Fig. 1b

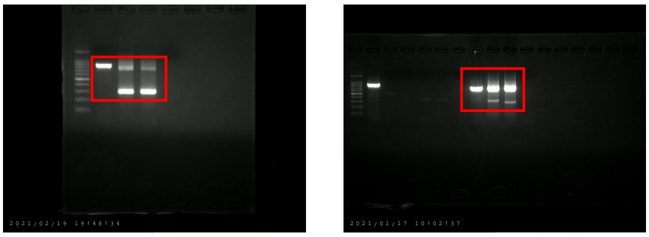

Fig. 1c

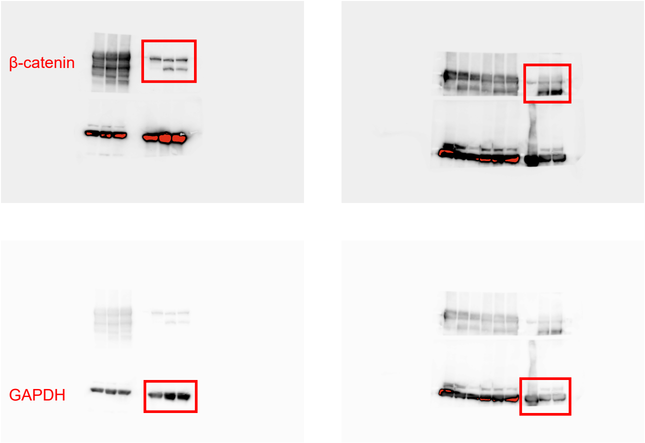

Fig. 2a

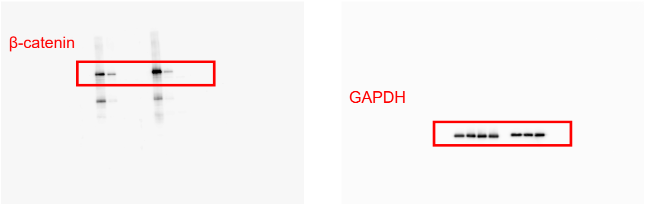

Fig. 2b

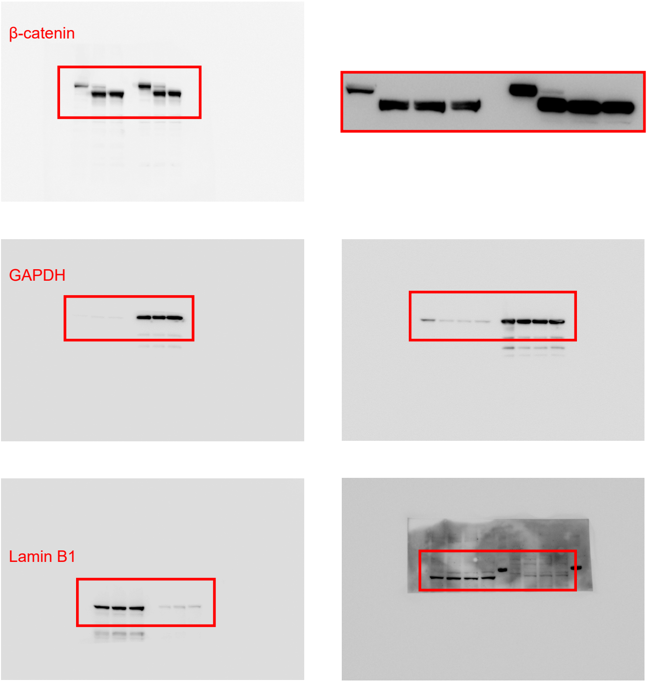

Supplementary Fig. 1

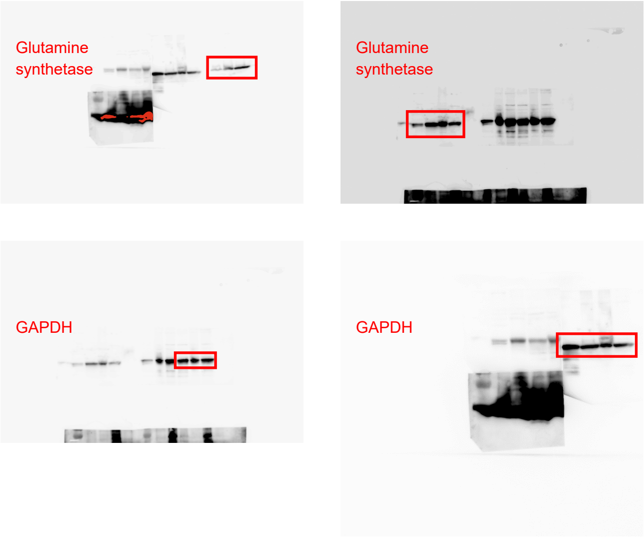

Supplementary Table 1. Genes upregulated in HCC samples with *CTNNB1* hotspot mutations.

Gene	log2 fold-change	*P*-value
RHBG	6.42	8.62E-33
NOTUM	6.38	1.35E-32
NKD1	6.01	1.76E-32
GLUL	3.97	2.12E-32
AXIN2	4.17	8.14E-32
LOC91834	2.79	6.69E-30
ODAM	7.62	3.16E-29
RGSL2	5.49	3.76E-29
TCF7	2.26	7.54E-29
SP5	3.16	1.28E-28
KCNU1	6.72	2.42E-28
ZNRF3	2.23	7.31E-28
LOC101929189	2.03	1.26E-27
LGR5	6.42	4.77E-27
AMACR	2.55	5.49E-26
SLC22A11	5.77	9.58E-26
SLC13A3	4.71	1.27E-25
SLC1A2	3.71	2.25E-25
AQP9	2.49	2.48E-25
FLJ37576	3.35	5.20E-25
LINC01124	2.38	7.06E-25
ALDH3A1	6.05	9.33E-25
RSPO2	4.08	1.01E-24
CTNNA2	7.25	4.29E-24
CLDN2	4.02	1.06E-23
HLF	2.37	1.38E-23

HEPACAM	7.84	2.04E-23
C1ORF64	4.93	3.37E-23
MGC47540	4.84	6.42E-23
LOC102724295	3.00	2.58E-22
RNF43	2.08	5.12E-22
JM27	8.33	7.36E-22
AQP6	4.39	4.98E-21
SEPT4	2.63	5.54E-21
UGT1A4	3.76	7.40E-21
RIPPLY1	2.36	1.93E-20
DKFZp547D155	7.39	2.20E-20
SLC12A1	5.43	2.83E-20
CYP1A1	5.93	5.93E-20
LOC201382	2.37	6.63E-20
SLC6A2	6.63	7.72E-20
CYP2E1	4.40	1.05E-19
INGAP	8.50	2.79E-19
LOC148709	2.03	3.83E-19
UGT1A5	3.17	1.30E-18
LPPR1	2.12	3.86E-18
MYRIP	2.23	4.36E-18
ALDH1L1	2.34	4.60E-18
CDH22	4.10	8.55E-18
SSTR1	2.49	9.72E-18
ACSL6	2.85	1.00E-17
PHYHIPL	2.13	1.23E-17
LRRC52	2.51	1.32E-17
SULT4A1	5.29	1.89E-17
IQCH	2.89	2.40E-17
SLC22A12	5.83	2.84E-17
CBLN4	3.20	3.61E-17

PRR5L	2.23	6.04E-17
C9ORF152	3.17	7.66E-17
TTC9	2.26	7.98E-17
UGT1A3	3.62	8.12E-17
DSG1	3.25	8.13E-17
ADRBK2	2.08	1.11E-16
GREB1	2.69	2.50E-16
HEPN1	7.70	3.07E-16
C15ORF43	2.39	3.88E-16
DKK4	4.68	8.73E-16
CYP3A3	5.60	9.28E-16
UBXN10	2.56	1.10E-15
ERK	2.93	1.33E-15
SLC45A2	2.59	1.71E-15
ZNF648	2.51	1.83E-15
RXRG	3.62	1.96E-15
LINC00238	2.61	2.10E-15
CYP1A2	5.34	2.58E-15
LINC00488	2.98	2.59E-15
ABHD1	2.31	3.58E-15
TNFRSF19	4.09	8.68E-15
TBX4	3.98	1.05E-14
MME	5.81	1.12E-14
GRPR	3.47	2.37E-14
ABCG2	2.21	2.85E-14
LPRS2	2.13	3.31E-14
RANBP3L	2.85	3.58E-14
CCDC170	2.26	3.81E-14
LGI3	3.20	6.13E-14
RHBDL3	3.84	9.78E-14
HPD	2.42	1.35E-13

UBE2QL1	2.39	1.47E-13
SLC14A2	2.50	4.43E-13
CST1	4.21	5.33E-13
BMP4	2.04	6.06E-13
SLCO1B3	4.90	1.11E-12
PART1	2.44	1.17E-12
LOC130913	2.05	1.94E-12
SLC16A11	2.16	2.41E-12
ADH1B	2.01	2.49E-12
FGF2	2.11	1.50E-11
LINC00176	2.58	1.80E-11
ADH4	2.23	2.38E-11
CA4	3.42	3.70E-11
ADCY8	3.34	4.07E-11
LOC196023	2.73	4.27E-11

Supplementary Table 2a. differentially expressed cytokine genes between the HuH7 cells with and without β-catenin signaling activation.

Gene	log2 fold-change	-log10 *P*-value
CXCL8	-3.56	23.00
CXCL6	-3.23	19.10
CXCL2	-2.34	14.11
CXCL10	-2.06	10.41
CCL20	-2.06	28.24
CXCL3	-2.05	9.15
CSF3	-2.05	5.84
CXCL1	-2.04	8.22
NAMPT	-1.98	25.98
CSF1	-1.77	9.17
SECTM1	-1.73	9.13
GDF15	-1.47	5.09
CTF1	-1.10	2.07
VEGFA	-1.08	5.89
CXCL5	-1.07	2.14
CKLF	-1.06	3.29
INHA	-0.99	2.26
IL12A	-0.95	1.65
CCL4	-0.74	1.12
YARS1	-0.70	3.97
CCL3	-0.60	0.87
IL7	-0.57	0.75
CXCL16	-0.47	1.02
INHBB	-0.46	0.65
VEGFD	-0.46	0.56

C5	-0.43	0.62
TRIP6	-0.39	0.74
MUC4	-0.22	0.26
SDCBP	-0.21	0.32
TYMP	-0.19	0.50
XCL1	-0.15	0.20
CNTF	-0.14	0.14
SPRED2	-0.11	0.29
ERBB2	-0.08	0.15
CCL22	-0.07	0.06
CX3CL1	0.03	0.08
SPRED1	0.04	0.07
AIMP1	0.06	0.15
PIK3R1	0.15	0.36
CCL15	0.19	0.21
SIVA1	0.19	0.27
ERAP1	0.23	0.76
MIF	0.28	0.46
TNFRSF11B	0.31	0.61
BABAM2	0.33	0.72
TGFB2	0.37	0.51
GLMN	0.38	0.90
ERBIN	0.43	1.74
CCL28	0.52	1.08
IL27	0.53	1.22
CDK5	0.88	4.19
BMP4	1.80	8.70

Supplementary Table 2b. differentially expressed cytokine genes between the 3H3 cells with and without β-catenin signaling activation.

Gene	log2 fold-change	-log10 *P*-value
Cxcl2	-2.79	9.76
Gdf15	-2.74	15.25
Cx3cl1	-1.96	19.94
Csf2	-1.45	3.13
Ccl7	-1.36	10.35
Vegfd	-1.19	3.19
Csf1	-1.16	6.51
Ccl20	-1.12	2.00
Inhba	-0.98	1.64
Csf3	-0.94	1.66
Vegfa	-0.94	3.10
Cxcl1	-0.86	1.70
Pf4	-0.85	1.47
Nampt	-0.79	9.09
Ccl28	-0.78	1.13
Ccl8	-0.77	1.25
Aimp1	-0.65	6.90
Cxcl12	-0.50	0.62
Cxcl5	-0.46	0.63
Cdk5	-0.46	1.95
Cxcl3	-0.35	0.43
Ccl25	-0.34	0.88
Il19	-0.34	0.40
Mif	-0.27	0.74
Trip6	-0.19	0.41

Ccl3	-0.16	0.17
Babam2	-0.15	0.46
Sdcbp	-0.14	0.37
Pik3r1	-0.14	0.49
Siva1	-0.11	0.31
Ccl17	-0.09	0.08
Cxcl16	0.01	0.02
Bmp4	0.06	0.11
Il7	0.08	0.10
Spred2	0.12	0.37
Inhbb	0.15	0.29
Spred1	0.15	0.42
Ctf1	0.16	0.24
Cklf	0.25	0.43
Ccl2	0.27	2.40
Muc4	0.27	0.35
Il1rn	0.35	0.53
Erbb2	0.44	3.81
Tgfb2	0.53	0.65
Glmn	0.60	2.83
Tymp	0.74	1.35
Erap1	1.14	11.30
Cntf	1.20	3.20
Erbin	1.31	6.99
Cxcl10	1.33	2.63

Supplementary Table 3. Methylation levels of downstream genes of the Wnt/β-catenin signaling pathway and cytokine genes.

Gene	*P*-value	Median β-value in HCC without *CTNNB1* mutation	Median β-value in HCC with *CTNNB1* mutation	Difference of median β-value
LGR5	6.14E-17	0.80	0.43	-0.37
RNF43	2.05E-11	0.32	0.18	-0.14
AXIN2	8.37E-28	0.60	0.15	-0.45
CCL20	0.090	0.82	0.78	-0.04
CXCL2	1.83E-05	0.50	0.69	0.20

Supplementary Table 4. Single guide RNA sequences.

Target site	Target sequence
Human CTNNB1 intron 2 1	5'-TTTCATCACTGAGCTAACCC-3'
Human CTNNB1 intron 2 2	5'-GAAAAGCAGAATGATAGCCA-3'
Human CTNNB1 intron 3	5'-CTTAGGTAAATGCTGAACTG-3'
Mouse Ctnnb1 intron 2 1	5'-TCCCTTCTGCACACTACCAC-3'
Mouse Ctnnb1 intron 2 2	5'-CACTCATTAGATCCCATCGG-3'
Mouse Ctnnb1 intron 3	5'-GCTGGTAAAGCATTTGTGTT-3'

Supplementary Table 5. Primers for detecting exon 3 skipping of β-catenin.

Gene	Forward and reverse primers	Size of product (bp)
Human CTNNB1	5'-TGCTCCATTTTCTGCTCACTC-3'<br>5'-GCATGCCCTCATCTAATGTCTC-3'	Wild-type: 836<br>Exon 3 skipping (in2-1/in3-1): 294<br>Exon 3 skipping (in2-2/in3-1): 294
Mouse Ctnnb1	5'-GCGTGGACAATGGCTACTCAAG-3'<br>5'-CTCTGAGCCCTAGTCATTGCAT-3'	Wild-type: 719<br>Exon 3 skipping (in2-1/in3-1): 307<br>Exon 3 skipping (in2-2/in3-1): 280

Supplementary Table 6. Primers for quantitative RT-PCR.

Gene	Forward and reverse primers	Size of product (bp)	Annealing temperature (°C)
Human LGR5	5'-CTCCCAGGTCTGGTGTGTTG-3'<br>5'-GAGGTCTAGGTAGGAGGTGAAG-3'	149	60
Human RNF43	5'-CATCAGCATCGTCAAGCTGGA-3'<br>5'-TTACCCCAGATCAACACCACT-3'	192	60
Human AXIN2	5'-TACACTCCTTATTGGGCGATCA-3'<br>5'-TTGGCTACTCGTAAAGTTTTGGT-3'	151	60
Human CCL20	5'-AAGTTGTCTGTGTGCGCAAATCC-3'<br>5'-CCATTCCAGAAAAGCCACAGTTTT-3'	107	60
Human CSF1	5'-TGAGACACCTCTCCAGTTGCTG-3'<br>5'-GCAATCAGGCTTGGTCACCACA-3'	151	60
Human CSF3	5'-GCTGCTTGAGCCAACTCCATA-3'<br>5'-GAACGCGGTACGACACCTC-3'	285	60
Human CXCL1	5'-GCGCCCAAACCGAAGTCATA-3'<br>5'-CTCAATCCTGCATCCCCCAT-3'	70	60
Human CXCL2	5'-GGCAGAAAGCTTGTCTCAACCC-3'<br>5'-CTCCTTCAGGAACAGCCACCAA-3'	127	60

Supplementary Table 6. Primers for quantitative RT-PCR.

Gene	Forward and reverse primers	Size of product (bp)	Annealing temperature (°C)
Human LGR5	5'-CTCCCAGGTCTGGTGTGTTG-3'<br>5'-GAGGTCTAGGTAGGAGGTGAAG-3'	149	60
Human RNF43	5'-CATCAGCATCGTCAAGCTGGA-3'<br>5'-TTACCCCAGATCAACACCACT-3'	192	60
Human AXIN2	5'-TACACTCCTTATTGGGCGATCA-3'<br>5'-TTGGCTACTCGTAAAGTTTTGGT-3'	151	60
Human CTNNB1	5'-CATCTACACAGTTTGATGCTGCT-3'<br>5'-GCAGTTTTGTCAGTTCAGGGA-3'	150	60
Human CCL20	5'-AAGTTGTCTGTGTGCGCAAATCC-3'<br>5'-CCATTCCAGAAAAGCCACAGTTTT-3'	107	60
Human CSF1	5'-TGAGACACCTCTCCAGTTGCTG-3'<br>5'-GCAATCAGGCTTGGTCACCACA-3'	151	60
Human CXCL2	5'-GGCAGAAAGCTTGTCTCAACCC-3'<br>5'-CTCCTTCAGGAACAGCCACCAA-3'	127	60
Human GDF15	5'-CAACCAGAGCTGGGAAGATTCG-3'<br>5'-CCCGAGAGATACGCAGGTGCA-3'	116	60
Mouse Lgr5	5'-CCTACTCGAAGACTTACCCAGT-3'<br>5'-GCATTGGGGTGAATGATAGCA-3'	165	60
Mouse Rnf43	5'-TCCGAAAGATCAGCAGAACAGA-3'<br>5'-GGACTGCATTAGCTTCCCTTC-3'	144	60
Mouse Axin2	5'-TGACTCTCCTTCCAGATCCCA-3'<br>5'-TGCCCACACTAGGCTGACA-3'	105	60

Mouse Ctnnb1	5'-TCCCATCCACGCAGTTTGAC-3'<br>5'-TCCTCATCGTTTAGCAGTTTTGT-3'	166	60
Mouse Ccl20	5'-GTGGGTTTCACAAGACAGATGGC-3'<br>5'-CCAGTTCTGCTTTGGATCAGCG-3'	105	60
Mouse Csf1	5'-GCCTCCTGTTCTACAAGTGGAAG-3'<br>5'-ACTGGCAGTTCCACCTGTCTGT-3'	124	60
Mouse Cxcl2	5'-CATCCAGAGCTTGAGTGTGACG-3'<br>5'-GGCTTCAGGGTCAAGGCAAACT-3'	103	60
Mouse Gdf15	5'-AGCCGAGAGGACTCGAACTCAG-3'<br>5'-GGTTGACGCGGAGTAGCAGCT-3'	106	60
Human/Mouse18S	5'-CGCTTCCTTACCTGGTTGAT-3'<br>5'-GAGCGACCAAAGGAACCATA-3'	137	60
